# Perspectives on the mechanism of pyroptosis after intracerebral hemorrhage

**DOI:** 10.3389/fimmu.2022.989503

**Published:** 2022-09-05

**Authors:** Dengpan Song, Chi-Tai Yeh, Jian Wang, Fuyou Guo

**Affiliations:** ^1^ Department of Neurosurgery, The First Affiliated Hospital of Zhengzhou University, Zhengzhou, China; ^2^ Department of Medical Research and Education, Shuang Ho Hospital, Taipei Medical University, New Taipei City, Taiwan; ^3^ Department of Pain Medicine, The First Affiliated Hospital of Zhengzhou University, Zhengzhou, China; ^4^ Department of Human Anatomy, School of Basic Medical Sciences, Zhengzhou University, Zhengzhou, China

**Keywords:** intracerebral hemorrhage, pyroptosis, inflammasome, secondary immune-inflammatory response, caspase-1, NLRP3

## Abstract

Intracerebral hemorrhage (ICH) is a highly harmful neurological disorder with high rates of mortality, disability, and recurrence. However, effective therapies are not currently available. Secondary immune injury and cell death are the leading causes of brain injury and a poor prognosis. Pyroptosis is a recently discovered form of programmed cell death that differs from apoptosis and necrosis and is mediated by gasdermin proteins. Pyroptosis is caused by multiple pathways that eventually form pores in the cell membrane, facilitating the release of inflammatory substances and causing the cell to rupture and die. Pyroptosis occurs in neurons, glial cells, and endothelial cells after ICH. Furthermore, pyroptosis causes cell death and releases inflammatory factors such as interleukin (IL)-1β and IL-18, leading to a secondary immune-inflammatory response and further brain damage. The NOD-like receptor protein 3 (NLRP3)/caspase-1/gasdermin D (GSDMD) pathway plays the most critical role in pyroptosis after ICH. Pyroptosis can be inhibited by directly targeting NLRP3 or its upstream molecules, or directly interfering with caspase-1 expression and GSDMD formation, thus significantly improving the prognosis of ICH. The present review discusses key pathological pathways and regulatory mechanisms of pyroptosis after ICH and suggests possible intervention strategies to mitigate pyroptosis and brain dysfunction after ICH.

## 1 Introduction

Intracerebral hemorrhage (ICH) is a common and devastating cerebrovascular disease with high morbidity and mortality rates, accounting for approximately 15% of all strokes and 50% of stroke-related mortality, and approximately 2.8 million deaths occur worldwide each year (1, 2). Following initial insult, secondary brain injury in patients with ICH contributes to poor outcomes, and effective therapy is still lacking (3, 4). Neuroinflammation plays a critical role in the pathogenesis of secondary brain injury (4, 5).

Pyroptosis is a recently discovered process that can lead to an inflammatory response. In contrast to common cell death pathways, such as necrosis, apoptosis, and autophagy, pyroptosis is characterized by unique expression patterns and mechanisms. Morphologically, pyroptosis exhibits features of both necrosis and apoptosis. Morphological changes include rupture of the necrosis-like cell membrane, pore formation, cell swelling, release of proinflammatory intracellular contents, nuclear condensation, and deoxyribonucleic acid (DNA) fragmentation. In contrast to apoptosis, pyroptosis is characterized by intact mitochondria and ‘balloon-shaped’ vesicle formation but not cytochrome c release (6, 7). Pore formation is a key factor in pyroptosis, and these pores allow the release of inflammatory cytokines such as interleukin (IL)-1β and IL-18. These inflammatory factors exacerbate the local inflammatory response, leading to neuronal death, destruction of the blood-brain barrier (BBB), and other types of secondary inflammatory damage in ICH (8).

Molecularly, pyroptosis is characterized by gasdermin-mediated cell death (9). The gasdermin protein family is made up of a pore-forming aminoterminal domain (N-terminal domain), which is automatically suppressed by the carboxyterminal domain (C-terminal domain) (9). Recent research suggests that gasdermin D (GSDMD) is one of the most important members. Activated caspase-1/4/5/11 effectively cleaves GSDMD at the aspartic acid site in the linker loop. Cleavage generates an N-terminal cleavage product (GSDMD-N) that binds to the plasma membrane and forms pores that trigger pyroptosis and release of the inflammatory cytokines IL-1β and IL-18 (10–13).

Normally, gasdermin is activated by two caspase-dependent pathways, including the canonical caspase-1 pathway and the noncanonical caspase-4/5/11 pathway (7). Caspase-1/4/5/11 are usually activated by inflammasomes or lipopolysaccharide (LPS). Inflammasomes are polyprotein complexes that are assembled from particular pattern recognition receptors (PRR) that recognize damage-associated molecular patterns (DAMP) and pathogen-associated molecular patterns (PAMP) in aberrant cellular settings. Toll-like receptors (TLR) and C-type lectin receptors (CLR) are transmembrane receptors, while retinoic acid-inducible gene (RIG) I-like receptors (RLR) and NOD-like receptors are cytosolic receptors (NLR) (14–16). Currently, the most widely studied inflammasome is the NOD-like receptor protein 3 (NLRP3) inflammasome, which has been shown to mediate the appearance of pyroptosis in a variety of diseases, including dilated cardiomyopathy, ischemic stroke, myocardial ischemia, ICH, inflammation and neoplastic diseases (17–24).

## 2 Mechanism of pyroptosis

### 2.1 Canonical pathway

**Figure 1:**
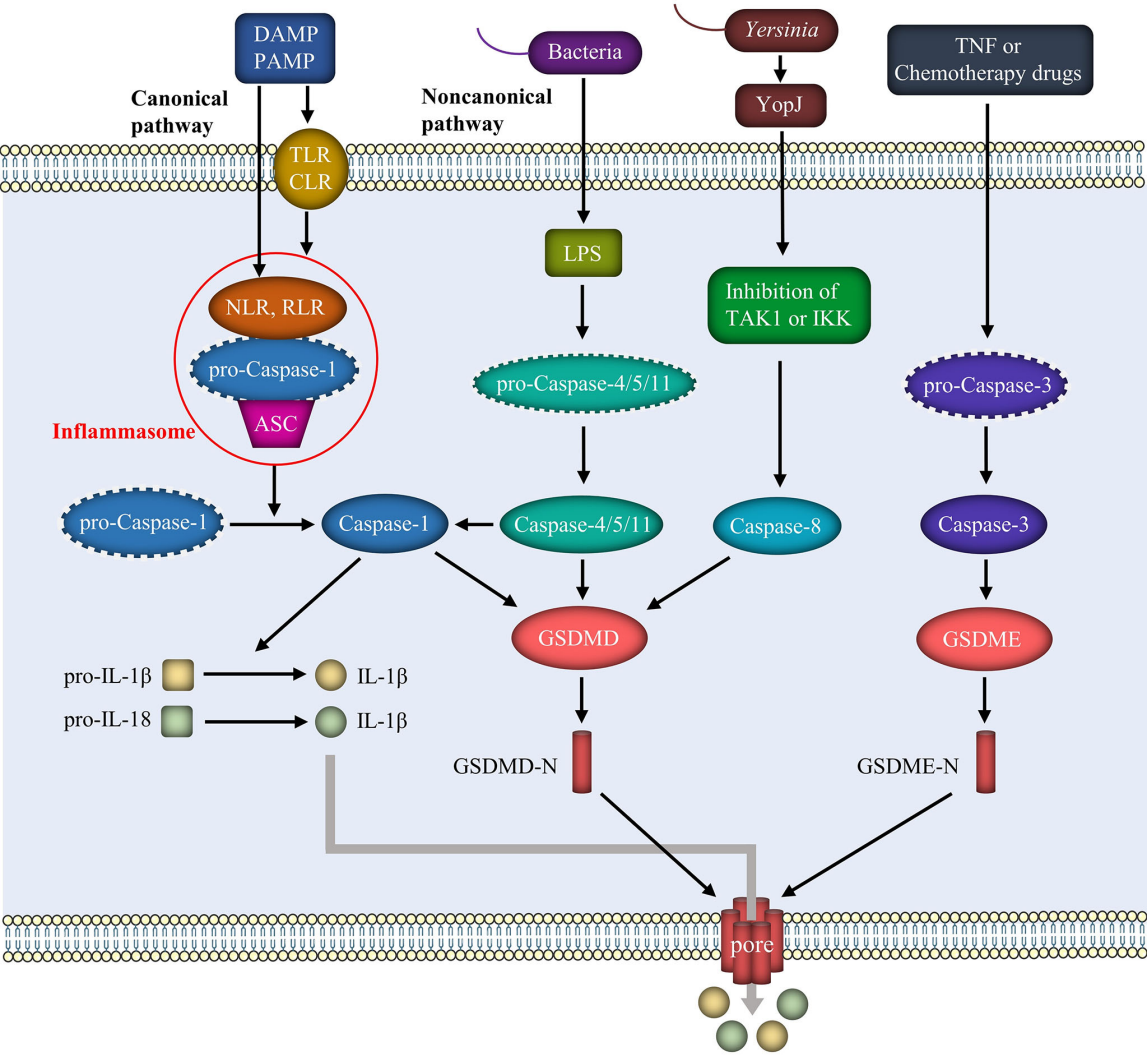
Mechanism of pyroptosis. The pathogenesis of pyroptosis includes mainly the canonical pathway mediated by the inflammasome and caspase-1, the noncanonical pathway mediated by LPS and caspase-4/5/11, and some other pathways involving caspase-8 and caspase-3. These pathways result in the cleavage of GSDMD or GSDME and the separation of the N-terminal fragments. GSDMD-N or GSDME-N forms pores in the cell membrane, leading to the release of inflammatory factors such as IL-1β and IL-18.

The classical pyroptosis pathway is mediated by caspase-1 (**Figure 1**). Pyroptosis signals trigger different cytoplasmic sensor proteins and promote the activation and release of different inflammasomes, as represented by NLRP3. Inflammasomes recruit pro-caspase-1 through an apoptosis-associated speck-like protein that contains a caspase recruitment domain (ASC), and the three factors bind and oligomerize to form inflammasomes (25). The activated inflammasome cleaves inactive pro-caspase-1 into mature caspase-1, which then converts pro-IL-1β and pro-IL-18 to mature IL-1β and IL-18, respectively (26). GSDMD is also cleaved by caspase-1, which separates its N-terminal pore-forming domain from the C-terminal repressor domain. The N-terminal fragment forms extensive gas-soluble pores on the cell membrane by dissolving phospholipids or cardiolipins, subsequently promoting the release of IL-1β and IL-18 and recruiting more extracellular inflammatory cytokines to cause inflammation (27, 28).

### 2.2 Noncanonical pathway

LPS released from Gram-negative bacteria, such as E. coli, binds directly to and activates the host immune receptors caspase-4/5 (in humans) and caspase-11 (in mice) through endocytosis. On the one hand, activated caspase-4/5/11 directly cleaves GSDMD, exerting the same effect as caspase-1 and leading to perforation of the cell membrane. On the other hand, in the presence of NLRP3 and the adapter protein ASC, activated caspase-4/5/11 interacts with caspase-1 to activate it, thus activating pyroptosis through the classic pathway. Furthermore, GSDMD-N oligomerization produces pores in the membrane after cleavage of GSDMD, as in the classical route, mediating the release of IL-1β and IL-18 into the extracellular space and causing an inflammatory cascade (29).

### 2.3 Other pathways

Tumor necrosis factor (TNF) or chemotherapy drugs activate caspase-3 to cleave gasdermin E (GSDME). The N-terminal fragment of GSDME perforates the cell membrane and causes pyroptosis (30). Furthermore, GSDMD-induced pyroptosis is regulated by caspase-8. Inhibition of TGF-β activated kinase-1 (TAK1) or inhibitor of κB kinase (IKK) by the Yersinia effector protein YopJ results in RIPK1- and caspase-8-dependent cleavage of GSDMD, resulting in pyroptosis (31). Pyroptosis is characterized by the release of IL-1β and IL-18. Caspase-8-induced pyroptosis occurs simultaneously with NLRP3 activation and IL-1β secretion (32). Furthermore, as a typical activator, ATP activates the NLRP3 inflammasome in macrophages, leading to caspase-1/GDSDMD-mediated pyroptosis (33). In addition to caspase-mediated pyroptosis, neutrophil serine endopeptidase activates GSDMD through the cleavage of L-cysteine and activates neutrophils *via* a mechanism independent of caspase-1/11 (34).

## 3 Potential mechanisms of pyroptosis after ICH

### 3.1 Pyroptosis caused by the classical pathway NLRP3/caspase-1 after ICH

**Figure 2:**
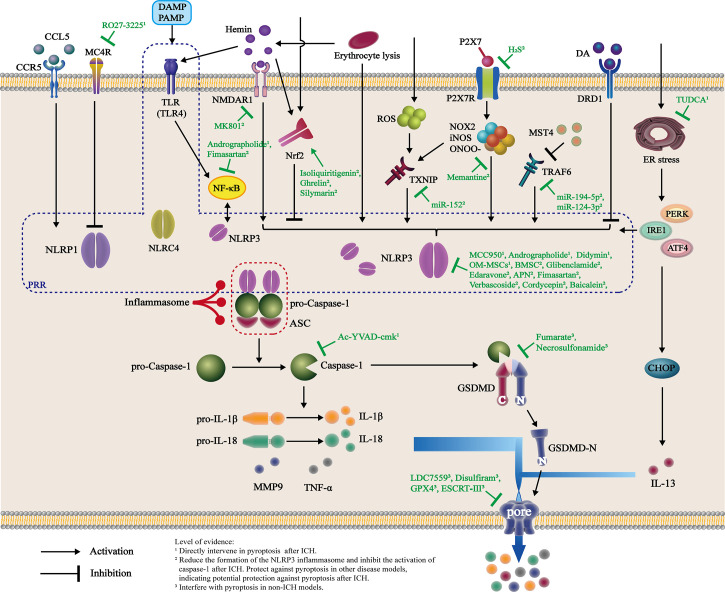
Activation and intervention of the pyroptosis pathway after ICH. Changes in the internal environment of the brain after ICH directly or indirectly activate PRR, as represented by NLRP3, through multiple pathways. These molecules recruit ASC and pro-caspase-1 and interact to form inflammasomes, which cleave pro-caspase-1 into active caspase-1. Caspase-1 cleaves both pro-IL-1β and pro-IL-18 into mature IL-1β and IL-18, respectively, and GSDMD to form GSDMD-N fragments. GSDMD-N binds to the plasma membrane and forms pores in the membrane, inducing the release of mature inflammatory factors such as IL-1β, IL-18, and TNF-α and subsequently recruiting more extracellular inflammatory cytokines and causing inflammation. Currently, animal- and cell-based experiments have shown that various drugs intervene in the formation of inflammasomes after ICH through different targets. Additionally, some candidate drugs are believed to directly interfere with pyroptosis after ICH, reducing the release of inflammatory factors and inhibiting neuroinflammation.

Caspase-1 activation is the key factor in the classical pathway of pyroptosis (**Figure 2**). The important roles of caspase-1 in pyroptosis and secondary immune inflammation after ICH have been documented. Several animal studies have confirmed that an increase in caspase-1 levels is observed at least 6 h after ICH in mice, and these levels generally peak at 24-72 h (35–37). Furthermore, by inhibiting caspase-1, IL-1β expression and matrix metalloproteinase-9 (MMP-9) activity are reduced, subsequently reducing BBB damage, improving nerve function, and attenuating brain edema. These effects might be mediated by the JNK pathway (38).

Activation of inflammasomes initiates the classical pyroptosis signaling pathway. To date, the best characterized inflammasome is assembled by NLRP3, which has been shown to be involved in the pathogenesis of atherosclerosis and brain injury caused by ischemia and ischemia/reperfusion (35, 39). NLRP3 is expressed in microglia and other types of brain cells, but this inflammasome component is not expressed after ICH. The current view is that microglial activation and polarization are important causes of secondary brain injury after ICH. ICH activates the NLRP3 inflammasome, and NLRP3 production increases biphasically, reaching a peak at 24 and 72 h after ICH (36). NLRP3 knockdown reduces brain edema and decreases myeloperoxidase (MPO) levels at 24 h and improves neurological function between 24 and 72 h after ICH (35). The expression of caspase-1 increases with increasing expression of NLRP3. Attenuation of ICH-induced NLRP3 overproduction results in decreases in caspase-1 and IL-1β expression, indicating that the NLRP3/caspase-1 signaling axis may mediate IL-1β overproduction after ICH (36). Similarly, the NLRP3 inflammasome amplifies the inflammatory response by releasing IL-1β and promoting neutrophil infiltration after ICH (35).

Direct evidence of changes in the morphology of pyroptotic cells is the formation of pores in the cell membrane. Li et al. directly confirmed the presence of neuronal pyroptosis after ICH using electron microscopy (40). Protecting neurons as much as possible is critical in the treatment of ICH and directly affects the prognosis. Although several studies have attempted to investigate the expression of inflammasome components and upstream regulators of canonical inflammasomes after ICH, few have explored the key protein GSDMD, which is downstream of canonical and noncanonical inflammasome activation. Furthermore, we propose to study the degree and changes in neuronal pyroptosis at different time points after ICH to verify whether pyroptosis plays a major role in secondary immune injury after ICH.

#### 3.1.1 Oxidative damage/NLRP3/caspase-1 pathway

Another interesting question is the possible mechanism underlying the formation of NLRP3 and the activation of caspase-1. Researchers have shown that the ATP-gated transmembrane cation channel purinergic 2X7 receptor (P2X7R) and the NLRP3 inflammasomes are activated after ICH. Silencing of the P2X7R gene substantially reduces brain edema and neurological impairment by suppressing the activation of the NLRP3 inflammasome and IL-1β/IL-18 production. Furthermore, elevated levels of NADPH oxidase 2 (NOX2) and inducible nitric oxide synthase (iNOS), as well as increased levels of their cytotoxic product peroxynitrite (ONOO−), which are important molecules associated with secondary immune injury after ICH, are markedly attenuated by treatment with a selective P2X7R inhibitor. This effect is accompanied by the downregulation of inflammasome components and IL-1β/IL-18 levels and decreased neutrophil activation. In particular, the degradation of ONOO− substantially reduces the activation of the inflammasome and the release of IL-1β/IL-18 (36, 41–43). These studies indicated that P2X7R is upstream of NLRP3 activation. P2X7R increases inflammatory progression and brain injury in ICH mice, potentially increasing IL-1β/IL-18 release and neutrophil infiltration through the NLRP3 inflammasome. ONOO−, a putative downstream signaling molecule of P2X7R, could play a key role in the activation of the NLRP3 inflammasome. Although P2X7 activation clearly peaks 24 h after ICH, ONOO−, NLRP3 and caspase-1 activation exhibit biphasic trends, with peaks occurring at 24 and 72 h. Therefore, we propose that the P2X7 pathway is only one of the causes of pyroptosis and manifests itself primarily as pyroptosis 24 h after ICH.

Thioredoxin binding protein (TXNIP), a member of the α-arrestin protein superfamily, is involved in inflammation and participates in the response of the reactive oxygen species (ROS)/TXNIP signaling pathway to oxidative stress together with ROS (44). TXNIP is involved in the induction of pyroptosis after diabetic nephropathy, alcoholic liver disease, and intestinal ischemia (45–47). TXNIP activates the NLRP3 inflammasome and facilitates the release of inflammatory cytokines after binding to NLRP3 (48). Zuo et al. found that thrombin release after ICH activates ROS/TXNIP/NLRP3 signaling in BV2 cells, thus activating the pyroptotic signaling pathway NLRP3/caspase-1/ASC, promoting the expression of IL-1β, IL-18 and TNF-α, and increasing the inflammatory response and cell death (49).

#### 3.1.2 Tumor necrosis factor receptor-associated factor 6 (TRAF6)/NLRP3 pathway

TRAF6 is a vital binding protein of the TNF and the TLR superfamily and is notably involved in innate immunity and acquired immunity. TRAF6 is involved in the pyroptosis process in cerebral ischemia, sepsis, acute pancreatitis, and other diseases and is associated with the manifestation of ICH (50–53). Mammalian sterile-20-like kinase 4 (MST4) is classified as a member of the GCK subfamily and is expressed at high levels in the thymus and brain (54, 55). MST4 also mediates cell growth and apoptosis, affecting cell migration, differentiation, and proliferation (54). As a negative regulator of inflammation, the kinase MST4 limits inflammatory responses through direct phosphorylation of the adapter TRAF6 (56–58). TRAF6 interacts with NLRP3 to promote activation of the NLRP3 inflammasome. TRAF6 overexpression after ICH increases NLRP3 inflammasome levels and other inflammatory factors, increasing cell death rate and reducing cell viability (56). However, since relevant studies are limited to activation of the NLRP3 inflammasome, it remains to study whether it causes pyroptosis through caspase-1 in ICH.

#### 3.1.3 Nuclear factor erythroid-2 related factor 2 (Nrf2)/NLRP3/caspase-1 pathway

Nrf2, an important transcription factor and the main regulator of cellular oxidative stress, induces antioxidant responses and increases the expression of downstream proteins, such as NAD(P)H:quinone oxidoreductase 1 (NQO1) and heme oxygenase 1 (HO-1) (59–63). Many studies have found that Nrf2 activation plays an essential role in protecting the brain from oxidative damage caused by ICH. Nrf2 activation in mice with ICH increases the expression of a series of antioxidant enzymes, detoxifying enzymes, and proteins, thus alleviating early brain injury after ICH and exerting a neuroprotective effect by activating Nrf2 (64, 65). These neuroprotective effects include improving neurological dysfunction, alleviating brain edema, reducing inflammatory cell infiltration, and accelerating hematoma clearance. Nrf2 knockout mice show more brain damage than control mice (66–72). Nrf2 inhibits pyroptosis by suppressing NLRP3 activation in microglia and vascular endothelial cells (73–74). Furthermore, Nrf2 activation inhibits expression of NLRP3/caspase-1/IL-1β and alleviates early brain damage after ICH by reducing neuronal death and the inflammatory response (75–77). Therefore, although direct evidence is not available, we postulate that Nrf2 participates in the mechanism that mediates pyroptosis after ICH.

#### 3.1.4 Dopamine D1 receptor (DRD1)/NLRP3/caspase-1 pathway

A previous study reported that the neurotransmitter dopamine inhibits the activation of the NLRP3 inflammasome *via* the DRD1 (78). Mechanisms involved in pyroptosis in Parkinson’s disease and spinal cord injury have been extensively investigated (79–82). Wang et al. reported that DRD1 activation decreases brain edema and improves neurobehaviors 24 and 72 h after ICH, respectively. Further mechanistic studies showed that DPD1 activation decreases IL-1β release and inhibits microglial activation and neutrophil infiltration by inhibiting pyroptosis and inflammation mediated by the NLRP3/caspase-1 pathway (83). However, there is no direct evidence that DRD1 is involved in mediating pyroptosis in the ICH model.

### 3.2 Pyroptosis caused by the NLRP1/caspase-1 and NLRC4/caspase-1 pathway after ICH

In addition to NLRP3, which has been widely studied, NLRP1 is another important component of the inflammasome in focal cell death after ICH. Jun et al. showed that the expression of the C-C chemokine ligand 5 (CCL5), the C-C chemokine receptor type 5 (CCR5), and NLRP1 in the brain is upregulated and reached a peak 24 h after ICH. CCR5 activation, which is mediated in part by CCR5/PKA/CREB/NLR1, enhances neuronal pyroptosis and neurological impairment in mice after ICH (4). Melocortin 4 receptor (MC4R), a seven-transmembrane G-protein coupled receptor, has previously been shown to exert anti-inflammatory and neuroprotective effects after traumatic brain injury and cerebral ischemia. Using the MC4R agonist RO27-3225, Chen et al. found that the MC4R/ASK1/JNK/p38 MAPK signaling pathway is also involved in NLRP1-dependent neuronal pyroptosis after ICH (37). The component of the NLRC4 inflammasome, another member of the NLR family, has recently been shown to play a critical role in pyroptosis and the resulting inflammation after ICH. Similarly to NLRP3 and NLRP1, NLRC4 amplifies inflammation by facilitating the processing of caspase-1, IL-1β, IL-18, and TNF-α. Furthermore, elimination of NLRC4 attenuates neuronal death, BBB damage, brain edema, and neurological deficiency 3 days after ICH (84). Therefore, it is essential to explore the role of the two pathways in pyroptosis after ICH and to see if they are related to the NLRP3/caspase-1 pathway.

### 3.3 Pyroptosis caused by erythrocyte lysis after ICH

Erythrocyte lysis is also involved in pyroptosis after ICH. The volume of the hematoma peaks and stabilizes from 24 to 72 h due to injury caused by primary rupture of the blood vessels and secondary destruction of the BBB and then begins to decrease (85–88). Furthermore, erythrocyte lysis and infiltration of neutrophils, macrophages, and microglia were observed at 24 h, and their levels peaked at 72 h, consistent with the increase in NLRP3 and caspase-1 levels described in other studies (89). The severity of perihematomal neuronal loss is proportional to the degree of erythrolysis. Furthermore, the addition of lysates to microglia increased the expression of NLRP3 and caspase-1 *in vitro*. On the one hand, lysis of red blood cells can induce oxidative stress and pyroptosis by causing ferroptosis (36, 90–92). Hemin, on the other hand, is a hemoglobin degradation product that is postulated to be a DAMP involved in the classical pyroptosis pathway (93). The results reported by Wen et al. showed that heme induces NLRP3-mediated inflammatory injury. Furthermore, the authors determined that the effect of heme was mediated by the N-methyl-D-aspartic acid receptor 1 (NMDAR1). NMDAR1 plays a pivotal role in hemin-induced NLRP3-mediated inflammatory damage through synergistic activation (94). Similarly, Lin et al. performed animal and cell studies and showed that hemin enhances microglial activation through TLR4, which in turn induces nuclear transcription factor-κB (NF-κB) activation through the MyD88/TRIF signaling pathway (95). Finally, the expression of IL-1β, IL-6, and TNF-α and the resulting inflammatory injury increases after ICH (95). When NF-κB, a downstream TLR molecule, is activated, it translocates to the nucleus and binds to DNA, ensuring full expression of NLRP3, pro-IL-1β, and pro-IL-18 mRNAs and proteins (96).

Another study provided that microRNA (miR)-223 is a potential negative regulator of NLRP3 formation using TargetScan (a program used to look for potential regulatory microRNAs). Inhibition of NLRP3 expression by miR-223 reduces microglial inflammation induced by erythrocyte lysis induced by erythrocyte lysis and neuronal injury after ICH in mice. Cell-based experiments also showed that miR-223 levels in microglia and ICH mice decrease after erythrocyte lysis (97). These studies indicate that erythrocyte lysis may be involved in the induction of cell death after ICH. Although the study proposes a possible role for pyroptosis, other groups have not replicated the results.

Furthermore, Nrf2 is also strongly associated with erythrocyte rupture products after ICH. Both free hemoglobin (Hb) released by rupture of the erythrocyte and free heme released by hemoglobin decomposition are cytotoxic and cause neuronal death and secondary inflammation (98–100). Haptoglobin (Hp) and hemopexin (Hx) interact with Hb and heme, respectively, to reduce their toxicity (101, 102). According to Zhao et al., Nrf2 activation increases Hp and Hx expression after ICH, exerting neuroprotective effects (71, 103). However, more studies are needed to determine whether this protective effect is related to pyroptosis.

### 3.4 Pyroptosis caused by endoplasmic reticulum (ER) stress after ICH

After ICH, other molecules or signaling pathways are activated and involved in pyroptosis. ICH leads to ER stress, which can induce neurological impairment through crosstalk with programmed cell death (PCD). Specifically, ER stress is caused by hypoxia and altered calcium homeostasis after ICH, mainly through the NLRP3/caspase-1 pathway. The ER stress-mediated NLRP3/caspase-1 pathway requires inositol-requiring protein-1 (IRE1) or protein kinase RNA-like endoplasmic reticulum kinase (PERK). ER stress induces pyroptosis (104). Furthermore, Chen et al. showed that ER stress through IL-13 causes neuronal pyroptosis after ICH. The AMP-dependent transcription factor-4 (ATF-4) and inositol-requiring kinase 1α (pIRE1α)/CCAAT-enhancer-binding protein homologous protein (CHOP) pathways, which are downstream targets of the PERK pathway, are also involved (85).

### 3.5 Endothelial cell pyrotosis and BBB destruction after ICH

Most studies on pyroptosis after ICH have focused on neuronal and glial cell death. The two significant pathological changes observed after ICH are neuronal death and BBB destruction. Xu et al. found that NLRP3 is activated in hemin-exposed cerebral microvascular endothelial bEnd3 cells and induces cell death. Furthermore, inhibition of NLRP3 after ICH alleviates brain edema and damage to the BBB. Based on this finding, the primary hematoma caused by the rupture of the blood vessel is likely to destroy the BBB by causing pyroptosis in the endothelium, forming a positive feedback loop that further exacerbates hemorrhage and secondary brain injury (7, 105). Suppression of caspase-1 improves angiogenesis and the prognosis of ischemia (106). Furthermore, Wu et al. found that caspase-1 activation and upregulation of IL-1β, JNK, and MMP-9 expression are also closely related to the disruption of the BBB (38). Pericytes and astrocytes, two other cells components of the BBB, should be investigated in future research on post-ICH pyroptosis.

### 3.6 Pyroptosis and microglial polarization after ICH

Microglia respond to acute brain injury by activating and developing classic M1-like (proinflammatory) or alternative M2-like (anti-inflammatory) phenotypes, a process termed polarization (107–109). Xia et al. performed cell-based experiments examining a mouse model of hyperlipidemic pancreatitis and found that macrophage M1 polarization is accompanied by activation of the caspase-1-mediated canonical pyroptosis pathway, leading to inflammation and damage to pancreatic tissue (110). Activation of the NLRP3 inflammasome has been shown in a variety of diseases to promote polarization of microglia/macrophages to the M1 phenotype (111–114). In individuals with ICH, selective inhibitors of the NLRP3 inflammasome reduce the polarization of M1 microglia around the hematoma, increase the number of M2 cells, and reduce neuroinflammation after ICH (61, 115). Selective inhibitors of caspase-1 exert a similar effect (116). Microglial polarization after ICH may be closely related to pyroptosis. Therefore, we hypothesized that M1 polarization of microglia after ICH is accompanied by activation of the pyroptosis pathway and that inflammatory factors released by the pyroptosis pathway likely induce subsequent microglial polarization, thus aggravating the secondary immune-inflammatory response. However, since no direct evidence exists, more experimental studies are needed to verify this hypothesis.

### 3.7 Other possible mechanisms to be confirmed

Chemotherapeutic drugs activate caspase-3 and induce pyroptosis by disrupting GSDME signaling in the treatment of lung, stomach, and colon cancer (30, 117–120). Caspase-3 is a well-recognized key molecule that causes cell apoptosis (121, 122). A conversion between apoptosis and pyroptosis occurs through a mechanism mediated by caspase-3/GSDME (121). Many studies have documented caspase-3 activation leading to apoptosis after ICH, and many signaling pathways have been shown to be involved (122–124). However, studies are needed to determine whether caspase-3/GSDME mediates pyroptosis after ICH in the future.

Recently, complement-mediated inflammation has also been reported to play a crucial role in ICH, including the secretion of IL-1β. Complement-induced ICH neuroinflammation depends on activation of NLRP3 (125). Although previous studies have shown that complement is widely involved in the pyroptosis observed in individuals with rheumatoid arthritis, membranous nephropathy, and infection (126–129), its role in pyroptosis after ICH and the specific underlying mechanism remain to be studied.

## 4 Potential interventions for pyroptosis after ICH

### 4.1 Regulation of the inflammasome

Inflammasome activation, such as NLRP3 activation, is associated with the appearance of pyroptosis, which results in the release of inflammatory mediators. Therefore, approaches targeting inflammasome activation are effective therapeutic strategies to alleviate pyroptosis and inflammatory injury after ICH (**Figure 2**, **Table 1**).

**Table 1:** Potential therapeutic drugs and targets for pyroptosis after ICH.

Drugs	Target	Level of evidence	Disease and model	Effect on pyoptosis	References
MCC950	NLRP3	1	Mouse ICH model with autologous blood or collagenase	Blocks NLRP3 activation, reduces IL-1β production, attenuates neurologic deficits and perihematomal brain edema, and improves BBB integrity	(130)
Didymin	NLRP3	1	Mouse ICH model with autologous blood, murine BV2 microglial cell line	Alleviates microglial pyroptosis and neuroinflammation through the NLRP3/Asc/caspase-1/GSDMD pathway by upregulating RKIP expression	(131)
Andrographolide	NF-κB, NLRP3	1	Rat ICH model with autologous blood, primary rat microglia, primary rat neurons	Inhibits the activation of NF-κB signaling pathway and assembly of the NLRP3 inflammasome, and decreases levels of IL-1β, IL-6, and TNF-α	(132)
Tauroursodeoxycholic acid (TUDCA)	ER stress, NLRP3	1	Mouse ICH model with collagenase	Inhibits ER stress through a mechanism involving NLRP3 and IL-13	(85)
OM-MSCs	NLRP3, caspase-1	1	Mouse ICH model with collagenase	Attenuates microglial activation and levels of NLRP3, caspase-1, IL-1β and TNF-α and reduces membrane pores	(40)
RO27-3225	MC4R/NLRP1	1	Mouse ICH model with collagenase	Suppresses NLRP1-dependent neuronal pyroptosis and improves neurological function, possibly mediated by activation of MC4R and inhibition of the ASK1/JNK/p38 MAPK pathways	(37)
Ac-YVAD-cmk	caspase-1	1	Mouse and rat ICH model with collagenase, primary rat microglia	Reduces caspase-1 activation and inhibits IL-1β production and maturation, but has no effect on NLRP3 expression	(116, 133)
Edaravone	NLRP3	2	Rat ICH model with autologous blood	Exhibits neuroprotection by suppressing NF-κB-dependent NLRP3 in microglia	(134)
Adiponectin (APN)	NLRP3	2	Rat ICH model with autologous blood	Decreases NLRP3 expression, decreases IL-1β and IL-18 production, alleviates neurological deficits, and reduces brain edema around the hematoma	(135)
MK801	NMDAR1/NLRP3	2	Mouse ICH model with hemin, N9 microglial cell line	Inhibits NMDAR1 to attenuate hemin-induced microglial activation and the production of NLRP3 and IL-1β	(94)
Verbascoside	NLRP3	2	Mouse ICH model with collagenase, primary mouse neurons	Inhibits inflammation and cell death and protects neurons by suppressing NLRP3 activation.	(136)
Cordycepin	NLRP3	2	Mouse ICH model with autologous blood	Inhibits inflammation and cell death and protects neurons by suppressing NLRP3 activation	(137)
Baicalein	NLRP3	2	Rat ICH model with collagenase	Inhibits inflammation and cell death and protects neurons by suppressing NLRP3 activation	(138)
Isoliquiritigenin	Nrf2/NLRP3	2	Rat ICH model with collagenase	Reduces ROS and NF-κB levels upon activation of the NLRP3 inflammasome pathway by triggering Nrf2-mediated antioxidant activity	(75)
Ghrelin	Nrf2/NLRP3	2	Mouse ICH model with autologous blood	Inhibits activation of the NLRP3 inflammasome and activates the Nrf2/ARE signaling pathway	(77)
Silymarin	Nrf2/NLRP3	2	Mouse ICH model with collagenase	Upregulates Nrf2/HO-1 signaling and inhibits NLRP3/caspase-1/IL-1β to prevent inflammation	(139)
MicroRNA-194-5p	TRAF6/NLRP3	2	Rat ICH model with collagenase	Reduces the interaction between TRAF6 and NLRP3 and inhibits the activation of the NLRP3 inflammasome	(140)
MicroRNA-124-3p	TRAF6/NLRP3	2	ICH patients, HMC3 cells	Reduces the interaction between TRAF6 and NLRP3 and inhibits the activation of NLRP3 inflammasomes	(141)
MicroRNA-152	TXNIP/NLRP3	2	Rat ICH model with collagenase, microglial BV2 cells, hippocampal neuronal HT22 cells	Inhibits TXNIP expression, blocks the TXNIP/NLRP3 interaction, inhibits activation of NLRP3-induced inflammasome and the expression of caspase-1, IL-1β, IL-18, and TNF-α	(142)
Hydrogen sulfide (H_2_S)	P2X7R/NLRP3	2	Rat ICH model with collagenase, primary rat microglia	Inhibits the P2X7R/NLRP3 inflammasome-associated neuroinflammatory response	(43)
Memantine	iNOS, ONOO^−^/NLRP3	2	Rat ICH model with collagenase	Inhibits the production of iNOS and ONOO, thus reducing the expression of NLRP3, IL-1β, and MMP9, and reduces neuronal death and brain edema to ameliorate neurological deficits	(143)
BMSC	NLRP3	2	Rat ICH model with collagenase, mouse BV2 microglia	Reduces brain edema and inhibits the inflammatory response through the miR-183-5p/PDCD4/NLRP3 pathway	(144)
Glibenclamide	NLRP3	2	Mouse ICH model with autologous blood	Maintains BBB integrity by inhibiting activation of the NLRP3 inflammasome in microvascular endothelial cells	(105)
Fimasartan	NF-κB, NLRP3	2	Rat ICH model with collagenase, mouse microglia BV2 cell line	Reduces the activation of the NLRP3/ASC/caspase-1 and NF-κB pathways	(145)
Glutathione peroxidase 4 (GPX4)	GSDMD	3	Mouse model of LPS-induced sepsis	Reduces lipid oxidation and limits the membrane localization of the N-terminal fragment of GSDMD	(146)
Dimethyl fumarate (DMF)	GSDMD	3	Bone marrow-derived macrophages (BMDM), mouse, MS patients	Inhibits GSDMD activation	(147)
Endosomal sorting complexes required for transport-III (ESCRT III)	GSDMD	3	BMDM, HeLa cells, HEK293T cells	Clears the broken cell membrane formed by pyroptosis and repairs the pores through pathways downstream of GSDMD activation	(148)
Necrosulfonamide (NSA)	GSDMD	3	HEK293T cells, THP-1 cells, murine macrophages, Mouse model of LPS-induced sepsis	Binds to GSDMD and inhibits GSDMD activation, which blocks pyroptosis and IL-1β release	(149)
LDC7559	GSDMD	3	Murine peritoneal neutrophils, HEK293T cells	Binds to GSDMD and suppresses pore formation	(150)
Disulfiram	GSDMD	3	THP-1 cells, HT-29 cells, HEK293T cells; mouse model of LPS-induced sepsis	Allows IL-1β and GSDMD processing but abrogates pore formation	(151)

1: Directly intervene in pyroptosis after ICH. 2: Reduce the formation of the NLRP3 inflammasome and inhibit the activation of caspase-1 after ICH. Protect against pyroptosis in other disease models, indicating potential protection against pyroptosis after ICH. 3: Interfere with pyroptosis in non-ICH models.

#### 4.1.1 Direct regulation of NLRP3

MCC950 is a potent, selective, small molecule NLRP3 inhibitor that blocks NLRP3 activation at nanomolar concentrations. MCC950 reduces IL-1β production, attenuates neurologic deficits and perihematomal brain edema, improves BBB integrity, and decreases pyroptosis after induction of ICH induction by injection of autologous blood or collagenase (130). Due to the high efficiency and specificity of MCC950, it is now widely used as a positive control to study the effect of NLRP3 inhibition. Edaravone and adiponectin (APN) exert similar effects to MCC950 in alleviating neurodegeneration and reducing the expression of mRNAs and proteins caspase-1, IL-1β and NF-κB (56, 130, 134, 135).

Didymin, a citrus dietary flavonoid, attenuates microglial activation and neutrophil infiltration by upregulating the expression of Raf kinase inhibitor protein (RKIP) and downregulating the expression of molecules related to pyroptosis, such as NLRP3, caspase-1, and GSDMD, and inflammatory cytokines, such as TNF-α and MPO, after ICH. Binding of RKIP to ASC interrupts the activation and assembly of the inflammasome (131). Andrographolide is one of the main active diterpenoid constituents of traditional Chinese medicine. It has been reported to exert anti-inflammatory effects mediated by the TLR4/NF-κB signaling pathway in treating liver inflammation and fibrosis (152, 153). Li et al. found that andrographolide improves neurobehavioral deficits and brain edema by inhibiting activation of the NF-κB signaling pathway and NLRP3 inflammasome assembly and reducing levels of IL-1β; andrographolide also improves ICH-induced microglial and neuronal pyroptosis. Furthermore, andrographolide significantly reduces the TNF-α and IL-6 levels by inhibiting the NF-κB signaling pathway (132). Other active ingredients in herbal medicine, such as verbascoside, cordycepin, and baicalein, inhibit inflammation and cell death and protect neurons following ICH by suppressing NLRP3 expression (136–138). Because of the common pharmacological actions, these drugs are likely to lessen pyroptosis after ICH.

Although the correlation between endothelial pyroptosis and BBB destruction after ICH has rarely been studied, Xu et al. have shown that glibenclamide maintains BBB integrity in an experimental ICH model by inhibiting the activation of the NLRP3 inflammasome in microvascular endothelial cells (105). Controlling and maintaining blood pressure after ICH are essential treatment effects. Controlling hypertension is vital to prevent the recurrence of ICH and to prevent ischemia and hypoxia, or even ischemic stroke caused by excessive decreases in blood pressure. Treatment with low doses of fimasartan, an angiotensin II receptor blocker, alleviates edema, improves neurological function, and alleviates secondary brain injury by significantly reducing activation of the NLRP3/ASC/caspase-1 and NF-κB pathways after ICH without changing mean blood pressure (145).

#### 4.1.2 Regulation of NLRP3 by cell-based therapy

Cell-based therapy is a promising avenue for ICH treatment. Treatments with mesenchymal stem cells (MSCs) of various sources have produced favorable neuroprotective and neurorestorative effects on ICH (154–156). Hypoxia-preconditioned ORs (OM-MSCs) attenuate microglial activation and reduce levels of IL-1β and TNF-α. Furthermore, hypoxia-preconditioned OM-MSCs mitigate pyroptosis by reducing the levels of proteins associated with pyroptosis in perihematomal brain tissues, decreasing the expression of microglial NLRP3 and caspase-1, and reducing membrane pores in microglia after ICH (40). Similarly, bone marrow mesenchymal stem cell (BMSC) transplantation contributes to attenuating neurological deficits after ICH (157). BMSCs improve neurological function, reduce brain edema, and inhibit the inflammatory response after diabetes-induced ICH through the miR-183-5p/PDCD4/NLRP3 pathway (144).

#### 4.1.3 Regulation of the Nrf2/NLRP3 pathway

Nrf2 regulation is closely related to NLRP3 expression. Intraperitoneal administration of isoliquiritigenin after ICH reduces early brain impairment and neurological deficits; The mechanisms underlying these effects involve negative regulation of ROS production and activation of NF-κB through the NLRP3 inflammasome pathway and activation of the Nrf2-mediated antioxidant system (75). Ghrelin, a peptide that participates in the brain-intestinal axis, has been shown to exert neuroprotective effects in many different neurological diseases, including subarachnoid hemorrhage (SAH), ischemic stroke, and traumatic brain injury (TBI) (158–161). Recent studies have shown that ghrelin protects against ICH-induced SBI by inhibiting activation of the NLRP3 inflammasome and activating the Nrf2/antioxidant response element (ARE) signaling pathway (77). Silymarin acts as a neuroprotective compound after ICH injury by activating Nrf2/HO-1 signaling and inhibiting NLRP3/caspase-1/IL-1β signaling to prevent inflammation (139).

#### 4.1.4 Regulation of the TRAF6/NLRP3 pathway

Studies of drugs targeting TRAF6 have also shown that these drugs regulate the expression of NLRP3. Wu et al. found that selective inhibition of MST4 expression by hesperidin increases the expression of NLRP3 inflammasome-related proteins, thus exacerbating neurological deficits and brain edema. Therefore, MST4 alleviates inflammatory progression and brain injury in mice with ICH, perhaps by reducing the activation of the NLRP3 inflammasome (56). Both microRNA-194-5p and microRNA-124-3p target TRAF6. Both molecules exert the same effect on TRAF6 through the same mechanism. Overexpression of miR-194-5p or miR-124-3p reduces the interaction between TRAF6 and NLRP3, thus inhibiting the activation of the NLRP3 inflammasome and reducing neuroinflammation during ICH (140, 141).

#### 4.1.5 Regulation of other molecules/NLRP3 pathway

MicroRNA-152 has been reported to possess anti-inflammatory properties, and its expression is downregulated after ICH. Overexpression of miR-152 in microglial BV2 cells reduces the hemin-induced inflammatory response and ROS generation, thus reducing the death of cocultured neuronal HT22 cells. Furthermore, overexpression of miR-152 by intracerebroventricular injection of a lentivirus in rats with ICH significantly alleviates brain edema, hematoma, and neurological deficits. Mechanistically, miR-152 significantly inhibits the expression of TXNIP induced by ICH, and its overexpression blocks the TXNIP/NLRP3 interaction, thus inhibiting the activation of the NLRP3 inflammasome and the expression of caspase-1, IL-1β, IL-18, and TNF-α to reduce neuroinflammation *in vivo* and *in vitro* (142).

Emerging studies have revealed the important physiological and pathophysiological roles of hydrogen sulfide (H_2_S) as a transmitter of NLRP3 inflammasome-associated neuroinflammation in the central nervous system. Exogenous administration of H_2_S reduces brain edema, microglial accumulation, and neurological deficits after ICH by inhibiting the neuroinflammatory response associated with the P2X7R/NLRP3 inflammasome, suggesting that it may be related to the pyroptosis pathway (43). Furthermore, Chen et al. also found that memantine inhibits the production of iNOS and ONOO−, thus reducing the expression of NLRP3, IL-1β and MMP9, reducing neuronal death and brain edema, and improving neurological deficits (143).

NMDAR1 is an important receptor that regulates NLRP3 after ICH. The NMDAR1 inhibitor MK801 has also been shown to attenuate hemin-induced microglial activation, which subsequently leads to a decrease in the production of NLRP3 and IL-1β in microglia (87). Tauroursodeoxycholic acid (TUDCA) alleviates neuronal pyroptosis by inhibiting ER stress after ICH through a mechanism involving NLRP3 and IL-13 (83). However, it remains to be explored whether they directly affect the pyroptosis process downstream of NLRP3.

#### 4.1.6 Regulation of the NLRP1 pathway

Few studies have been conducted on non-NLRP3 inflammasomes. As mentioned above, NLRP1, NLRC4, and some TLRs may also be involved in the process of pyroptosis after ICH. RO27-3225, an MC4R agonist, was found to suppress NLRP1-dependent neuronal pyroptosis and improved neurological function, possibly mediated by activation of the MC4R and inhibition of the ASK1/JNK/p38 MAPK signaling pathway after ICH (37).

### 4.2 Regulation of caspase-1 and interleukin signaling

Bypassing inflammasomes and directly interfering with molecules downstream of the focal death pathway have also been shown to be effective treatments for ICH. Ac-YVAD-cmk, a well-known selective inhibitor of caspase-1, reduces caspase-1 activation and inhibits IL-1β production and maturation, but it does not have an effect on NLRP3 expression. Ac-YVAD-cmk inhibits pyroptosis, decreases inflammatory factor secretion or activation, and affects microglial polarization and aggregation, resulting in improvements in neurological function and behavioral performance and amelioration of brain edema after ICH. Neuroprotection is associated with decreased expression of IL-1β, IL-18, and MMP-9 and inhibition of ZO-1 degradation (38, 116, 133).

### 4.3 Regulation of GSDMD cleavage and cell membrane repair

The cleavage and activation of gasdermin is the most direct cause of membrane pores and the release of inflammatory factors that induce the inflammatory response. Therefore, direct inhibition of gasdermin proteins or recombining C-terminal and N-terminal fragments could also inhibit pyroptosis. Kang et al. found that glutathione peroxidase 4 (GPX4) decreases lipid oxidation and thus limits membrane localization of the N-terminal fragment of GSDMD and negatively regulates GSDMD-mediated pyroptosis in LPS-induced sepsis (146). According to Fitzgerald et al., fumarate, an intermediate in the tricarboxylic acid cycle, succinylates cysteine in GSDMD, inhibits its interaction with and activation of cysteine proteases, and ultimately inhibits the occurrence of pyroptosis. Related therapeutics, such as tecfidera, have been used to treat multiple sclerosis (MS) (147). Furthermore, the endosomal sorting complexes required for transport-III (ESCRT III)-dependent exocytosis can clear the broken cell membrane caused by pyroptosis and repair the pores in the cell membrane after GSDMD activation, thus strongly inhibiting pyroptosis and IL-1β release (148). Necrosulfonamide (NSA) was originally identified as a cysteine-reactive drug that targets the mixed-lineage kinase domain-like protein (MLKL) to inhibit necroptosis (162, 163). Furthermore, NSA binds directly to GSDMD and inhibits its activation. NSA blocks pyroptosis and IL-1β release in human and murine monocytes/macrophages without interfering with inflammasome formation and GSDME-mediated cell death (149, 162). LDC7559 inhibits the formation of neutrophil extracellular traps (NET), presumably by binding to GSDMD and suppressing pore formation (150). Disulfiram also covalently modifies human/mouse Cys191/Cys192 in GSDMD to block pore formation. Interestingly, disulfiram still allows IL-1β and GSDMD processing, but abrogates pore formation, thus preventing IL-1β release and pyroptosis in LPS-induced sepsis (151). Various methods have been developed to curb pyroptosis by regulating GSDMD and repairing pores. It should be emphasized that current studies mainly focus on the upstream molecular regulatory mechanisms of pyroptosis after ICH. No studies have explored the role and mechanism of related drugs targeting GSDMD regulation and cell membrane repair in pyroptosis in ICH. Due to their similar mechanisms of action, these treatments are likely to play equally important roles in the pyroptosis process after ICH and therefore should be explored in detail.

## 5 Conclusions and future directions

ICH still threatens life due to high morbidity and mortality. Currently, many original studies have focused on the research hotspot of pyroptosis. The mechanism of pyroptosis in various diseases, such as sepsis and tumors, has gradually become known due to recent studies. However, basic research on the role of pyroptosis in ICH and related interventions for ICH is lacking. Although there is increasing evidence to support the link between pyroptosis and ICH, there is no detailed summary of the available knowledge. This review focuses on ICH and comprehensively and systematically introduces recent research findings, mechanisms of pyroptosis after ICH, and potential intervention strategies. Pyroptosis in neurons, microglia, and endothelial cells after ICH is closely related to impairments in neural function, activation of immune inflammation, and destruction of the BBB. The NLRP3/caspase-1/GSDMD pathway induces pyroptosis after ICH. Specifically, changes in the brain microenvironment after ICH activate various NLRP3 upstream molecules, including NMDAR1, TXNIP, and so on. It induces NLRP3 and contributes to the assembly of inflammasomes, thus activating caspase-1. In addition to increasing the release of IL-1β, IL-18, TNF-α and other inflammatory factors, caspase-1 also cleaved GSDMD to release GSDMD-N, which formed pores in the cell membrane and promoted the release of inflammatory factors. On the one hand, released inflammatory factors damage normal tissues through the immune response; on the other hand, they can also reactivate the above pyroptosis reaction, forming a positive feedback effect, and further aggravating brain injury. It would also be interesting to investigate whether pyroptosis plays a role in mediating the interaction between the immune system and the autonomic nervous system after ICH (164, 165).

Evidence indicates that pyroptosis could play an essential role in ICH injury and that the targeted intervention has achieved positive results in preclinical research. However, it is unclear whether pyroptosis is the leading cause of secondary brain injury after ICH. Although we have proposed the above assumptions based on preclinical studies, most studies focused on the NLRP3 inflammasome. Therapies targeting downstream caspase-1 and GSDMD of pyroptosis have not been tested. Therefore, the specific mechanism of pyroptosis after ICH needs further exploration. We also suggest that future studies on pyroptosis after ICH should be more complete. In addition to NLRP3, it is unclear whether NLRP3 and NLRC4 play the same critical role in the pyroptosis process of pyroptosis after ICH.

Meanwhile, caspase-1/11 and GSDMD are specific or universal markers of pyroptosis, and their intervention effect after ICH needs clarification. We propose that caspase-1 might have a connecting role in the pyroptosis process after ICH and suggest exploring and developing potential drugs targeting it. Erythrocyte breakdown after ICH might be one of the causes of pyroptosis. We hypothesize that controlled erythrocyte lysis is critical against pyroptosis. Investigating this possibility is worthwhile. Currently, relevant compounds may have multiple targets, but they do not target pyroptosis-related molecules. It is worth investigating whether the main target of these drugs is primarily pyroptosis.

**Figure 3:**
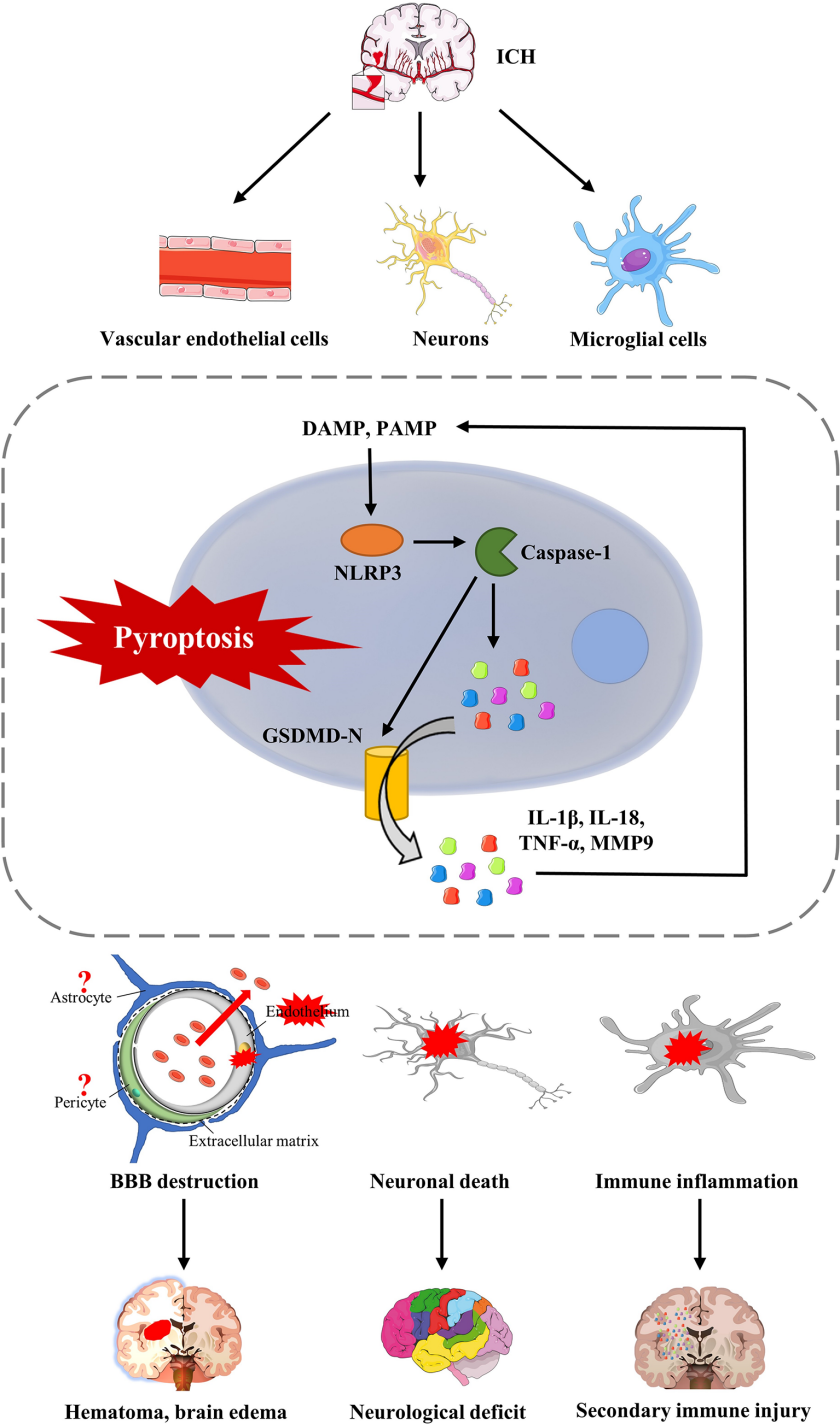
The consequences of pyrotosis after ICH. Pyroptosis of cerebral microvascular endothelial cells after ICH can lead to disruption of the BBB, further aggravated by the formation of hematoma and brain edema. Furthermore, pyroptosis, which occurs in neurons, leads to the death of functional neurons, which is a fundamental cause of neurological impairment and complications after ICH. Furthermore, pyroptosis of microglia after ICH will release various inflammatory factors, such as IL-1β, IL-18, and TNF-α, and recruit more extracellular inflammatory factors, leading to secondary immune injury.

Like other modes of cell death, pyroptosis ultimately leads to brain cell death and the resulting histopathology and functional dysfunction (**Figure 3**). Pyroptosis of cerebral microvascular endothelial cells after ICH leads to disruption of the BBB. It further aggravates the formation of hematoma and brain edema. The hematoma mechanically compresses the surrounding normal brain tissue. Exudation and lysis of the erythrocyte can further enhance pyroptosis, as described above. It remains unclear whether pericytes and astrocytes undergo pyroptosis and participate in the disruption of the BBB after ICH. Primary brain damage may be due to pyroptosis in many neurons, leading to neurological deficits and a poor prognosis. The most crucial characteristic of pyroptosis, which is different from other modes of cell death, is that many inflammatory factors produced in the pyroptosis process permeate brain tissue by forming cell membrane pores. Pyroptosis of microglia after ICH may be the most critical initiator. The release of inflammatory factors, such as IL-1β, IL-18 and TNF-α, leads to secondary immune injury. The excessive immune response will lead to more neuronal death, increased neurological deficits, and a worse prognosis. Therefore, we should focus on the vital role of microglia in pyroptosis after ICH and further explore whether there is a causal relationship between microglia activation and polarization and pyroptosis.

In conclusion, current research shows that inhibition of pyroptosis can alleviate tissue damage after ICH. A breakthrough in the treatment of ICH could be achieved by targeting pyroptosis, which could eventually be an effective strategy to improve the prognosis of patients. However, most current studies focus only on the underlying mechanism; in addition, most studies use cell and animal models rather than clinical trials. Currently, no relevant clinical research has been completed. Therefore, basic scientists and clinicians must carefully design translational research and clinical trials on pyroptosis to test novel therapeutic strategies to treat ICH.

## Author contributions

FG, JW, and DS conceptualized the manuscript outline. DS performed the literature search, wrote the manuscript, and designed the figures. FG, JW, and C-TY provided comments and proof-reading. All authors read and approved the final manuscript.

## Funding

This work was supported by the National Key Research and Development Program of China (2021YFE0204700).

## Conflict of interest

The authors declare that the research was conducted in the absence of any commercial or financial relationships that could be construed as a potential conflict of interest.

## Publisher’s note

All claims expressed in this article are solely those of the authors and do not necessarily represent those of their affiliated organizations, or those of the publisher, the editors and the reviewers. Any product that may be evaluated in this article, or claim that may be made by its manufacturer, is not guaranteed or endorsed by the publisher.

## Glossary

**Table:**

ICH	intracerebral hemorrhage
BBB	blood-brain barrier
GSDMD	gasdermin D
LPS	lipopolysaccharide
PRR	pattern recognition receptors
DAMP	damage-associated molecular patterns
PAMP	pathogen-associated molecular patterns
TLR	toll-like receptors
CLR	C-type lectin receptors
RLR	retinoic acid-inducible gene-I-like receptors
NLR	NOD-like receptors are cytosolic receptors
NLRP3	NOD-like receptor protein 3
NLRC4	NOD-like receptors domain containing 4
ASC	apoptosis-associated speck-like protein containing a caspase recruitment domain
IL	interleukin
TNF	tumor necrosis factor
GSDME	gasdermin E
TAK1	TGF-βactivated kinase-1
IKK	inhibitor of κB kinase
MMP-9	matrix metalloproteinase-9
MPO	myeloperoxidase
P2X7R	purinergic 2X7 receptor
NOX2	NADPH oxidase 2
iNOS	inducible nitric oxide synthase
TRAF6	tumor necrosis factor receptor-associated factor 6
MST4	mammalian sterile-20-like kinase 4
Nrf2	nuclear factor erythroid-2-related factor 2
NQO1	NAD(P)H quinone oxidoreductase 1
HO-1	heme oxygenase-1
TXNIP	thioredoxin binding protein
ROS	reactive oxygen species
DRD1	Dopamine D1 receptor
CCL5	C-C chemokine ligand 5
CCR5	C-C chemokine receptor type 5
MC4R	melocortin 4 receptor
NMDAR1	N-methyl-D-aspartic acid receptor 1
NF-kB	nuclear transcription factor-kB
Hb	hemoglobin
Hp	Haptoglobin
Hx	hemopexin
ER	endoplasmic reticulum
PCD	programmed cell death
TBI	traumatic brain injury
H_2_S	hydrogen sulfide
miR	microRNA
RKIP	Raf kinase inhibitor protein
TUDCA	tauroursodeoxycholic acid
MSCs	mesenchymal stem cells
BMDMs	Bone marrow-derived macrophages
GPX4	glutathione peroxidase 4
DMF	dimethyl fumarate
MS	multiple sclerosis
ESCRT III	endosomal sorting complexes required for transport-III
MLKL	mixed-lineage kinase domain-like protein
NET	neutrophil extracellular traps.
